# Protecting the properties of monolayer MoS_2_ on silicon based substrates with an atomically thin buffer

**DOI:** 10.1038/srep20890

**Published:** 2016-02-12

**Authors:** Michael K. L. Man, Skylar Deckoff-Jones, Andrew Winchester, Guangsha Shi, Gautam Gupta, Aditya D. Mohite, Swastik Kar, Emmanouil Kioupakis, Saikat Talapatra, Keshav M. Dani

**Affiliations:** 1 Femtosecond Spectroscopy Unit, Okinawa Institute of Science and Technology Graduate University, Onna, Okinawa, 904-0495 Japan; 2 Department of Materials Science and Engineering, University of Michigan, 2106 H. H. Dow Bldg, 2300 Hayward St., Ann Arbor, MI 48109, USA; 3 Material Synthesis and Integrated Devices, MPA-11, Los Alamos National Laboratory, Los Alamos, NM 87545, USA; 4 Department of Physics, Northeastern University, Boston, Massachusetts 02115, USA; 5 Department of Physics, Southern Illinois University Carbondale, Carbondale, Illinois 62901, USA

## Abstract

Semiconducting 2D materials, like transition metal dichalcogenides (TMDs), have gained much attention for their potential in opto-electronic devices, valleytronic schemes, and semi-conducting to metallic phase engineering. However, like graphene and other atomically thin materials, they lose key properties when placed on a substrate like silicon, including quenching of photoluminescence, distorted crystalline structure, and rough surface morphology. The ability to protect these properties of monolayer TMDs, such as molybdenum disulfide (MoS_2_), on standard Si-based substrates, will enable their use in opto-electronic devices and scientific investigations. Here we show that an atomically thin buffer layer of hexagonal-boron nitride (hBN) protects the range of key opto-electronic, structural, and morphological properties of monolayer MoS_2_ on Si-based substrates. The hBN buffer restores sharp diffraction patterns, improves monolayer flatness by nearly two-orders of magnitude, and causes over an order of magnitude enhancement in photoluminescence, compared to bare Si and SiO_2_ substrates. Our demonstration provides a way of integrating MoS_2_ and other 2D monolayers onto standard Si-substrates, thus furthering their technological applications and scientific investigations.

In recent years, transition metal dichalcogenides (TMDs) have gained much attention as semiconducting van der Waals materials that can be obtained in monolayer form^1^,^2^,^3^,^4^,^5^. Analogous to the technological importance of graphene for its electronic properties, the presence of a direct band gap in the visible part of the spectrum^2^,^3^, as well as large optical absorption^6^,^7^,^8^,^9^ and photoconductivity^10^,^11^,^12^ in TMDs, particularly monolayer MoS_2_, have cemented their importance in opto-electronic devices and applications, such as solar cell devices^9^,^13^,^14^,^15^, photodetectors^16^,^17^,^18^, and other flexible opto-electronics^19^. Recent demonstrations have suggested the utility of MoS_2_ in spin and valleytronic devices^20^,^21^,^22^, as well as nanoelectronic devices with metallic and semiconducting phases^23^,^24^,^25^. However, like most atomically thin 2D materials, when placed on a substrate, interactions impact key opto-electronic, structural, and physical properties of monolayer MoS_2_, preventing its full realization for potential technological applications. For example, quenching of photoluminescence on silicon^2^,^3^ inhibits its utility in silicon-integrated valleytronic and spintronic devices. Similarly, diffuse electron diffraction patterns on Si and SiO_2_^26^ suggest a distorted crystalline structure or poor surface morphology, which impacts device performance^27^. Substrate interactions also inhibit the study of phase-engineered MoS_2_ opto-electronic devices^23^,^24^, where crystalline structure provides an important characterization tool for the different metallic and semiconducting phases. Thus the ability to integrate monolayer MoS_2_ onto standard Si-devices, while simultaneously protecting a wide range of its key properties, is important for scientific and technological applications of MoS_2_.

Given the practical requirements of substrates for 2D materials, a number of studies have explored substrate interactions for MoS_2_ and other 2D crystals^2^,^3^,^4^,^26^,^28^,^29^,^30^,^31^. In MoS_2_, electron probes of the surface structure noted the lack of a sharp diffraction pattern for monolayer samples on Si and SiO_2_ for mechanically exfoliated, as well as in chemical vapor deposition (CVD) grown crystals^26^. Atomic force microscopy studies have shown a strong dependence of surface roughness on the underlying substrate^29^. Similarly, reports of photoluminescence quenching on Si^2^,^3^ and SiO_2_^4^,^28^ were attributed to charge transfer from the substrate to monolayer MoS_2_^4^,^28^. Other bulk substrates^4^ such as hBN, LaAlO_3_ and SrTiO_3_ were shown to protect photoluminescence, by preventing such a charge transfer, but these lack easy-integration into standard electronics processing technologies. On the other hand, atomically thin sheets of hBN on Si-based substrates can easily be integrated into Si-based electronic devices and have been shown to improve electron mobilities^6^,^32^,^33^. However, the ability of an atomically thin buffer layer to protect optical properties by preventing charge transfer^34^, or to restore sharp crystallinity by screening substrate interactions, remains unexplored. In general, a suitable solution to integrate monolayer MoS_2_ into standard Si-based processing technologies, while protecting the wide range of opto-electronic, structural and morphological properties of MoS_2_ remains elusive.

In this article, we demonstrate that an atomically thin buffer layer of hBN simultaneously protects the range of key opto-electronic, structural and morphological properties of monolayer MoS_2_ on Si-based substrates (Fig. 1). Using microprobe-Low Energy Electron Diffraction (μ-LEED), we show that monolayer MoS_2_ on bare Si and SiO_2_ substrates exhibits a diffuse diffraction pattern and a warped surface morphology. On the other hand, the atomically thin hBN buffer layer restores sharp diffraction patterns and results in an extremely flat 2D morphology, with over an order of magnitude less surface roughness. Using micro-photoluminescence and micro-Raman, we further show that the photoluminescence and Raman with the hBN buffer is over two orders of magnitude larger than on bare Si, and is enhanced compared to even suspended samples. This ability to protect a wide range of key properties of monolayer MoS_2_ on Si-based substrates thus enables sophisticated applications such as valleytronics^20^,^21^,^22^ and phase-engineered devices^23^,^24^,^25^, where the preservation of multiple intrinsic properties of MoS_2_ are simultaneously required.

To study the effect of an atomically thin hBN buffer layer between monolayer MoS_2_ and Si based substrates, we prepared a variety of mechanically exfoliated samples using a viscoelastic stamp^35^. First, mono- to few-layer samples of MoS_2_ were directly exfoliated on bare Si and SiO_2_. These were compared to monolayer flakes suspended on a grid of 2.5 μm holes on Si. Finally, atomically thin buffer layers of hBN with 1–5 nm thickness were first exfoliated onto SiO_2_/Si, followed by exfoliation of larger MoS_2_ flakes on top. These structures were annealed at 200 °C for a few hours in an ultrahigh vacuum chamber. Further details of sample preparation are presented in the Methods Section. Optical images of the different samples are shown in Fig. 2(a–d), with the regions of monolayer MoS_2_ identified by the dotted white line.

To study the structural and surface morphological properties of the MoS_2_ flakes, we used a Low Energy Electron Microscope (LEEM) capable of measuring spatially resolved electron diffraction patterns with sub-micron resolution. Opto-electronic properties were studied using commercial micro-PL and micro-Raman setups. Details of experimental conditions are presented in the Methods Section.

Electron diffraction obtained from monolayer MoS_2_ placed on Si and SiO_2_ substrates (Fig. 2e,f) shows a significantly weakened and diffuse diffraction pattern compared to suspended samples (Fig. 2g), indicating structural disorder or surface roughness. Similarly, PL maps (Fig. 2i–l) of the MoS_2_ flakes on the different substrates allow us to distinguish the regions of monolayer, but show a significantly weakened PL on the Si and SiO_2_ substrates compared to the suspended sample (Fig. 2m). We also observe a quenched Raman signal on Si (Fig. 2n). This weak electron diffraction, photoluminescence, and Raman on Si-based substrates limit the utility and integrability of MoS_2_ in standard opto-electronic devices. In contrast, the presence of an atomically thin buffer layer of hBN protects the range of key structural and opto-electronic properties of MoS_2_. As seen in Fig. 2(h,m,n), a protective buffer layer of hBN recovers a sharp diffraction pattern, enhanced photoluminescence and Raman from monolayer MoS_2_ by reducing substrate interactions.

To further explore the role of hBN in protecting these properties in MoS_2_ and its potential applicability to other 2D monolayers, we study electron diffraction as a function of incident electron energy. The protective layer of hBN can be as thin as ~1 nm, as shown in Figs S1 and S2. In particular, we measure the broadening of the diffracted electron beam for different MoS_2_ samples (Fig. 3). This allows us to distinguish between two important mechanisms that cause diffuse electron diffraction – a) lattice distortions or defects in the crystal structure, or b) surface roughness of the 2D crystal. In the case of lattice distortions, the broadening in the diffuse diffraction pattern is independent of incident electron energy^36^. On the other hand, surface roughness leads to a linear increase in the FWHM of the diffuse diffraction pattern versus incident electron energy^37^,^38^. In this case, the roughness causes the scattered electron wave to propagate in directions deviating from the specular direction, with increasing deviations for increasing incident electron energy, as illustrated in Fig. 3(c).

By plotting the FWHM of the (00) diffraction beam (σ) versus incident electron wave vector k_⊥_ for monolayer MoS_2_ on different substrates (Fig. 3d), the mechanism for diffraction broadening can be elucidated. (Details of the fitting process are in the Methods Section). For the Si and SiO_2_ substrates, we clearly see a linearly increasing σ superimposed on the resonant features resulting from structural effect in the samples. This indicates a high degree of roughness of the MoS_2_ monolayer on top of Si and SiO_2_. In comparison, one sees a much smaller σ, with a smaller linear increase in the suspended sample, indicating that the suspended sample exhibits much lower, but non-negligible surface roughness. In contrast, virtually no broadening of the diffraction pattern is detected for MoS_2_ on hBN with almost no increase in σ as a function of the incident electron energy, indicating an extremely flat 2D monolayer.

Figure 4 compares the surface roughness for MoS_2_ flakes of different thickness on both SiO_2_ and hBN. Monolayer of MoS_2_ shows the highest roughness on SiO_2_ while films of greater thickness rapidly relax to form flat films. On the other hand, MoS_2_ films of all thicknesses show fairly flat topologies on hBN. While our results typically explore samples of few-nm thick hBN, Fig. S1 shows that we obtain a sharp diffraction pattern and an extremely flat 2D monolayer even for a ~1 nm thick hBN buffer layer. Assuming that broadening of diffraction beams is caused by a Gaussian distribution of surface normal, surface roughness of the MoS_2_ film can be quantified by the relation Δθ = Δk_‖_/2 k_⊥_, with a corresponding magnitude of 4.0°, 0.58°, and 0.48° for 1 ML, 2 ML, and 3 ML MoS_2_ on SiO_2_, respectively. We see a comparable value of 4.0° for 1 ML MoS_2_ on bare Si. For suspended monolayer MoS_2_, surface roughness can similarly be quantified as 0.7°, in agreement with previous experimental^39^ and theoretical^27^ results. In contrast, the roughness on hBN is given by 0.08°, reduced by more than an order of magnitude compared to SiO_2_, Si or even the suspended samples. From our LEED measurements and analysis, we see that the surface roughness of monolayer MoS_2_ on few-nm thick hBN is comparable to bulk MoS_2_, which is consistent with previous AFM studies where the surface roughness of monolayer MoS_2_ on bulk hBN was comparable to bulk MoS_2_^29^.

In order to further study the interaction of MoS_2_ with Si and hBN/Si substrates, we performed density functional theory calculations (Fig. 5) to understand our experimental observations (See Methods section for details). For 2 ML MoS_2_ directly deposited on Si (111), the bottom MoS_2_ layer is significantly distorted with an in-plane S–S distance fluctuation of 1.98% (Fig. 5a). We attribute the distortion to bond formation between the bottom S and the surface Si atoms, as the shortest Si–S distance is 2.39 Å, which is comparable to the calculated Si–Si bond length (2.36 Å) and only 10% larger than the Si–S bond length in SiS_2_ (2.13 Å)^40^. This implies that any Si surface roughness is transferred to the MoS_2_ ML. The top MoS_2_ layer of the 2 ML structure (and any subsequent layer farther away from the interface) is largely unaffected by interactions with the substrate because of weak van der Waals bonds between MoS_2_ layers, which are much weaker than intralayer bonds. This result agrees with the experimental observation that the roughness of MoS_2_ decreases for increasing numbers of MLs. In contrast, the MoS_2_ ML distortions are one order of magnitude smaller (0.13% at most) if deposited on the 4 ML hBN/Si (111) surface (Fig. 5b). This is also attributed to the weak van der Waals bonds between layers and agrees with the flatness of MoS_2_ on the hBN/Si surface observed in experiment. In addition, hBN interacts weakly with the substrate, as evident from the 2 × 1 reconstruction of the Si(111) surface^41^, which shows that the Si surface atoms interact weakly with hBN. The smallest Si–N and Si–B distances are 3.21 Å and 3.28 Å, respectively, which are similar to the van der Waals bond length between hBN layers (3.35 Å)^42^. Moreover, the hBN layers are not distorted by the Si substrate. Therefore, as a result of the weak van der Waals interaction between all adjacent layers in the MoS_2_/hBN/Si heterostructure, the hBN layers and the MoS_2_ ML on top are weakly distorted, which agrees with the observed low roughness of both hBN and MoS_2_ when deposited sequentially on Si (111).

In the triad of 2D materials – graphene as a metal, transition metal dichalcogenides as semiconductors, and hBN as an insulator, the role of hBN in protecting the electronic^32^,^33^,^43^ and structural properties^33^,^43^ of graphene has been previously reported. These properties also extend to the protection of other 2D monolayer properties, such as MoS_2_, using bulk hBN, due to its inert chemical form, lack of dangling bonds, and flatness^44^,^45^. Our results show that hBN’s protective capabilities can be achieved even with just a few atomic layers. We see that even with a few atomically thin layers of hBN on SiO_2_/Si (Fig. S2), we obtain a stable, flat platform, which thereby prevents the overlying 2D monolayer from conforming to the roughness of the substrate.

Besides providing a stable, flat platform, atomically thin hBN needs to also screen interactions arising from the underlying Si-based substrate, which is not a consideration for bulk hBN substrates. DFT calculations for MoS_2_ sitting directly atop Si, show a large distribution in the bond lengths in MoS_2_, due to substrate interactions. Such distortions contribute to structural and morphological deformities observed via the diffuse crystalline patterns and quenched Raman signals, as well as modification of the electronic structure and optical responses^13^,^46^. In contrast, in the presence of an atomically thin layer of hBN, the bonds in MoS_2_ are minimally distorted, with hBN acting as a barrier layer to minimize interactions with the Si substrate. Thus the ability of few-layer hBN to provide a flat platform and to minimize substrate interactions, provides insight into the observation of morphologically flat and undistorted crystals of monolayer MoS_2_.

In addition to improvements in surface flatness and lattice distortion playing a role in protecting the opto-electronic properties^6^,^32^,^33^, such as the PL enhancement seen here, we also expect that the few nm layer of hBN acts as a barrier to charge transfer from the SiO_2_/Si substrate. This has been previously attributed as an important factor in PL quenching^4^,^47^. Lastly, we also note here the importance of annealing and cleaning the MoS_2_/hBN heterostructure, which results in further improvements in the PL (Fig. S1), presumably due to removal of impurities and trapped states at the MoS_2_/hBN interface^48^. Thus, overall we expect that the PL enhancement seen here is due to a combination of multiple factors – decreased surface roughness and lattice distortion due to reduced substrate interaction in the presence of the hBN buffer; the action of the hBN buffer as a barrier to charge transfer from the substrate; and decreased impurity/trapped states after annealing of the heterostructure sample.

In conclusion, we have shown that an atomically thin buffer layer of hBN simultaneously protects a range of key properties of monolayer MoS_2_ on Si-based substrates. The atomically-thin hBN buffer allows for easy integration of monolayer MoS_2_ into standard electronics devices, thus enhancing its utility in valleytronics, phase engineered nanoscale electronics, and other opto-electronic devices. Our results also have obvious implications for incorporating other 2D monolayers into standard Si-based devices, while protecting their opto-electronic, structural and morphological properties.

## Methods

### Sample preparation

MoS_2_ flakes are prepared by exfoliation of MoS_2_ single crystals supplied by Manchester Nanomaterials with the well-known scotch tape technique^49^. We transferred the MoS_2_ flakes onto different substrates and created the MoS_2_/hBN/SiO_2_ heterostructure by an all-dry transfer method using a viscoelastic stamp (GelFilm from Gel-Pak) and a home-built micro-manipulator^35^. For MoS_2_ on SiO_2_, we used SiO_2_/Si substrates with 300 nm of thermal oxide, which was first cleaned in an ultrasonic bath with acetone and then rinsed by methanol. For MoS_2_ on Si, an oxide free Si surface is prepared by flash cleaning of the Si(111) wafer to a temperature close to the melting point of Si in the ultrahigh vacuum chamber (UHV) in LEEM. Sample cleanliness and oxide removal is confirmed by appearance of sharp (7 × 7) reconstructed surface with low energy electron diffraction^50^. For suspended MoS_2_ flakes, hole of diameters of 2.5 μm and depth of 6 μm were drilled on the SiO_2_/Si substrate by focused ion beam (FEI Helios NanoLab G3 UC). Before MoS_2_ transfer, substrates with holes were first annealed in UHV chamber at 200 °C to remove Ga contamination from the ion milling process. For MoS_2_/hBN/SiO_2_ heterostructures, SiO_2_/Si substrates were first cleaned by wet chemical method, as described above. hBN flakes of approximately few nm in thickness were prepared by exfoliation of an hBN single crystal (Manchester Nanomaterials) and then transferred onto the SiO_2_/Si substrate using the GelFilm. Thin hBN flakes of uniform thickness were identified by AFM (Agilent 5500 AFM). MoS_2_ flakes were transferred and positioned on top of the flat hBN flake using another GelFilm. The samples were then cleaned and annealed in an UHV chamber in preparation for μ-LEED and surface characterization measurements as described below. The cleaning and annealing process was also critical to observing the significant, order-of-magnitude enhancements in PL (Fig. S1b).

### μ-LEED and Surface Roughness Characterization

Crystallinity and surface roughness of the MoS_2_ flakes were investigated using a low energy electron microscope (LEEM) (Elmitec SPELEEM), which enables high resolution imaging of large sample areas (>100 μm) at resolution better than 10 nm, as well as microprobe-diffraction imaging. This allows access of structural information in sub-micron selected areas of less than 250 nm. LEEM uses very low energy electrons of few eV to image surfaces; hence it is extremely sensitive to surface contamination. Samples introduced in the LEEM imaging chamber are cleaned either by mild annealing at 200 °C for several hours or by illumination of electron beams which remove contamination from local area^38^. Samples cleaned by either method give similar diffraction patterns, revealing the crystallinity of the MoS_2_ samples. We do not observe any degradation of crystallinity by performing any further annealing or electron illumination. Charging of the sample surface occurs during imaging, in particular, when the MoS_2_ samples are on top of insulating substrates such as SiO_2_. In our studies, we are able to eliminate detrimental charging effects by simultaneously illuminating the sample with an intense UV pulsed laser beam, which generates enough photoexcited carriers to neutralize any charging effects. Similarly, by using a very small incident electron beam of less than 250 nm in diameter during diffraction pattern imaging (μ-LEED), very little charging of the surface was observed. This could again be compensated by a very weak photon beam if needed.

### Optical Characterization

Raman and photoluminescence characterization of the MoS_2_ flakes were performed with a Nanofinder 30 (Tokyo instruments) with an excitation laser wavelength of 532 nm. Spatially resolved spot sizes of 0.5 μm were typically achieved in the measurement. PL spectra are taken at power of 1 mW and an exposure time of 10 s. Raman spectra are taken at a power of 2 mW, and an exposure time of 10 s with 5 accumulations.

### First-Principles Calculations

Density Functional Theory calculations were performed using the Vienna Ab initio Simulation Package^51^ with projector-augmented waves^52^ and a cutoff of 350 eV. We used the optB86b-vdW exchange-correlation functional^53^ to account for the van der Waals interaction between the layered materials, which yields accurate lattice parameters for MoS_2_^54^. Simulation supercells containing 230 (2 ML MoS_2_ on Si) and 443 (1 ML MoS_2_ on 4 ML hBN on Si) atoms were used to simulate the layer-substrate interactions. The positions of the Si atoms on the opposite side of the slab were fixed to the bulk values during atomic relaxation, and dangling bonds were passivated with H atoms. The structures are relaxed until the force on each atom is smaller than 8 × 10^−3^ eV/Å.

## Additional Information

**How to cite this article**: Man, M. K. L. *et al.* Protecting the properties of monolayer MoS_2_ on silicon based substrates with an atomically thin buffer. *Sci. Rep.* **6**, 20890; doi: 10.1038/srep20890 (2016).

## Supplementary Material

Supplementary Information

## Figures and Tables

**Figure 1:**
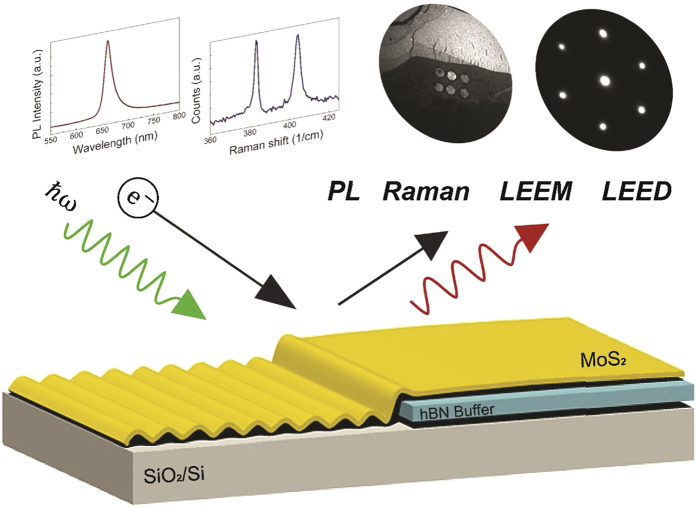
Schematic of the characterization of monolayer MoS_2_ with an atomically thin hBN buffer on Si-based substrates. We characterize a range of key properties including photoluminescence (PL), Raman, Low Energy Electron Microscope (LEEM) images, Low Energy Electron Diffraction (LEED), and surface morphology of the monolayer with and without the buffer. On the bare substrate, we observe diffuse electron diffraction, quenched PL, quenched Raman, and a rough surface. The presence of the hBN buffer results in an order of magnitude stronger PL, Raman, sharper electron diffraction, and flat surface morphologies.

**Figure 2:**
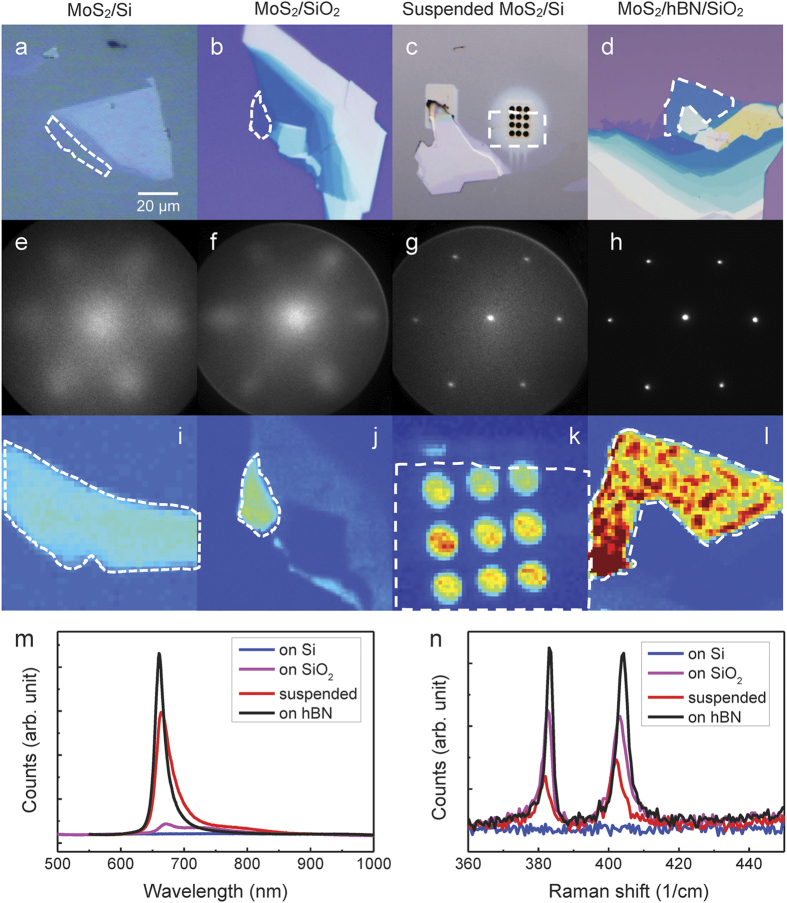
Optical, μ-LEED pattern, PL and Raman spectra of monolayer MoS_2_ on different substrates. (**a–d)** shows optical images of the MoS_2_ flakes, with monolayer areas outlined (white dashed line). In (**c**), the black circles are 2.5 μm diameter holes drilled into the Si substrate by focused ion beam with a suspended monolayer MoS_2_ on top. (**e–h**) shows the μ-LEED pattern of monolayer MoS_2_ using an electron beam of 250 nm diameter at 50 eV. Relatively weak and diffuse diffraction patterns are obtained for monolayer MoS_2_ on top of Si and SiO_2_, whereas for suspended MoS_2_ and MoS_2_ on top of hBN buffer, sharp, pristine diffraction patterns are observed. (**i–l**) are intensity maps of the PL peak with the color-scale optimized individually for visibility. Comparison of PL signal strength and Raman spectra of different samples are given in (**m**,**n**), respectively. The monolayer resting on the hBN buffer shows more than an order of magnitude increase in PL over the monolayer on bare Si or SiO_2_.

**Figure 3:**
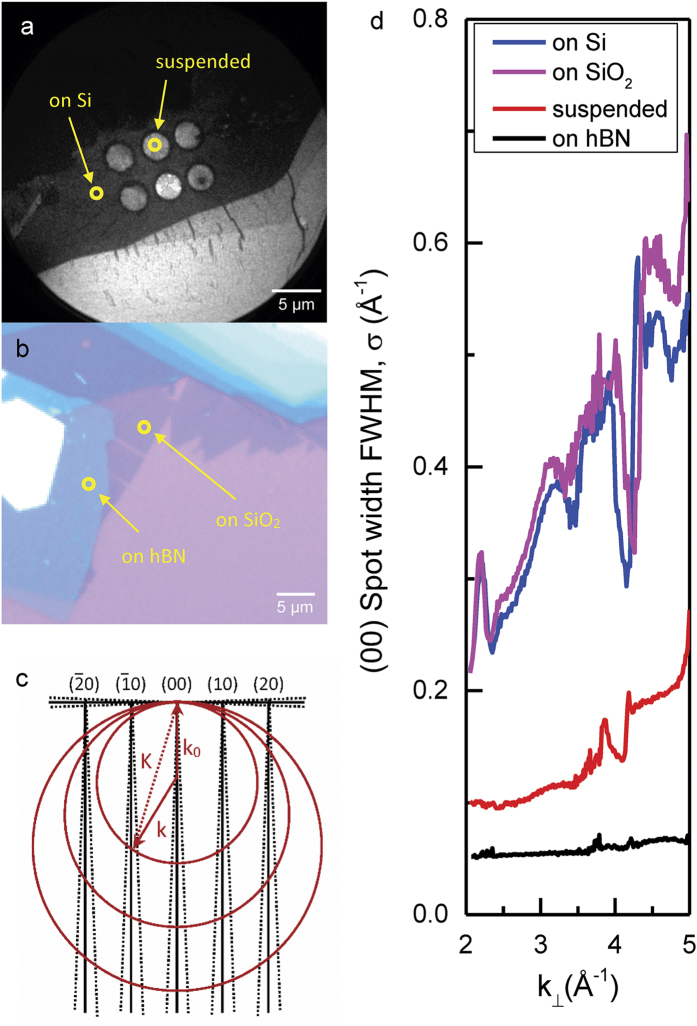
Comparing surface roughness of monolayer MoS_2_ on different substrates. (**a**) LEEM images of MoS_2_ flakes sitting on top of a holey Si substrate. (**b**) Optical image of an MoS_2_ flake on SiO_2_ with hBN buffer in between. Markers in (**a,b**) indicate positions where detailed LEED spot profile analyses were done. (**c**) explains the mechanism that causes broadening of the observed LEED diffraction pattern. A rough surface produces diffraction beams which spread out as a cones perpendicular to the sample surface, causing increased broadening with increasing incident electron energy. (**d**) The FWHM of the specular (00) diffraction beam–σ, with respect to the incident electron wave vector k_⊥_. Superimposed on the resonant features due to few-layer effects, the overall linear increase in σ indicates a high degree of surface roughness of the MoS_2_ on bare Si and SiO_2_. A smaller slope in suspended MoS_2_ shows a much reduced, but non-zero surface roughness in suspended samples, while the hBN buffer results in yet another order of magnitude decrease in surface roughness.

**Figure 4:**
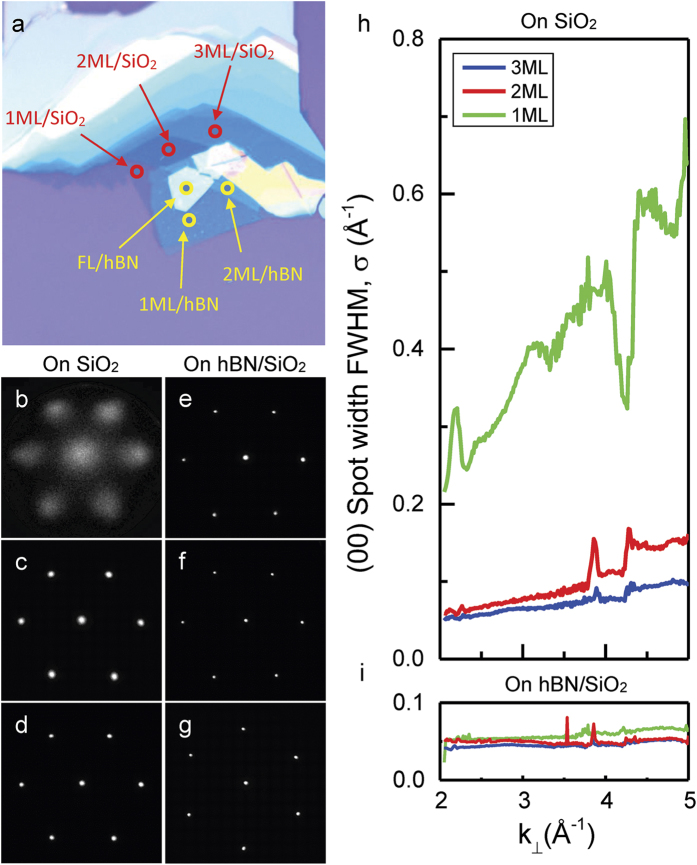
Surface roughness for mono-, bi- and few-layer MoS_2_ on SiO_2_ and on hBN buffer. (**a**) Optical image of a MoS_2_ flake lying partially on top of SiO_2_ and partially on top of a hBN buffer. (**b–d**) shows the μ-LEED pattern of 1 ML, 2 ML and 3 ML MoS_2_ on SiO_2_ and (**e–g**) shows the μ-LEED pattern of 1 ML, 2 ML and few layers MoS_2_ on hBN. All LEED are taken at 50 eV. (**h**) The FWHM of the specular (00) diffraction beam–σ, versus incident electron wave vector k_⊥_. The slope of the overall linear increase of σ versus k_⊥_ shows the decreasing surface roughness from mono- to bi- to few-layer MoS_2_ on SiO_2_. On the other hand, MoS_2_ on hBN displays negligible amount of beam broadening for all thickness.

**Figure 5:**
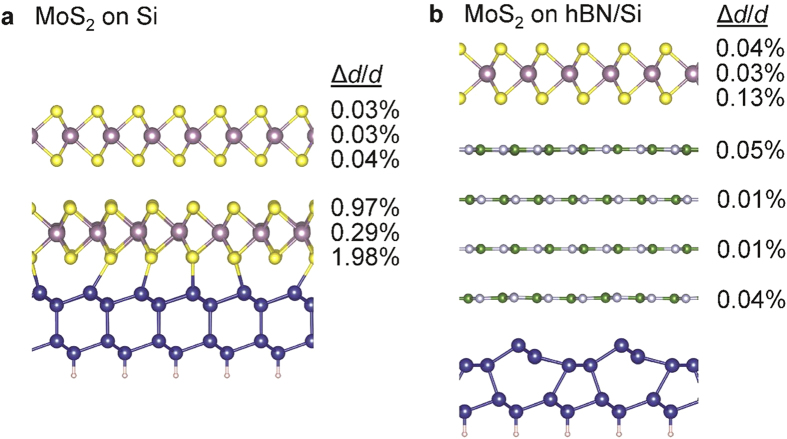
Density functional theory calculations of atomic interactions between MoS_2_ and Si or hBN/Si surfaces. Relative variation of the in-plane lattice constant (Δ*d*/*d*) for each atomic layer due to MoS_2_-substrate interactions for (**a**) 2 ML MoS_2_ on Si (111) and (**b**) 1 ML MoS_2_ on 4 ML hBN on Si (111). (**a**) The bottom MoS_2_ layer interacts strongly with dangling bonds on the Si surface and gets distorted, while the structure of the top MoS_2_ layer is largely unaffected due to the weak van der Waals bond between the two layers. Si–S atoms with a distance shorter than 2.6 Å are connected with bonds for illustration. (**b**) Inserting 4 MLs of hBN between MoS_2_ and the Si substrate significantly suppresses their interaction and distortion, as evident by the small fluctuation of in-plane bond lengths of MoS_2_ and the 2 × 1 surface reconstruction of Si, due to weak van der Waals bonds between adjacent layers.
