# Supplementary material for: Protecting the properties of monolayer MoS2 on silicon based substrates with an atomically thin buffer
Source: Sci Rep. 2016 Feb 12;6:20890. doi: 10.1038/srep20890 (PMC4751437; doi:10.1038/srep20890)
Supplement: Supplementary Information [file srep20890-s1.pdf]

Protecting the properties of monolayer MoS_2 on silicon based substrates with an atomically thin buffer

Michael K.L. Man[1], Skylar Deckoff-Jones[1], A. Winchester[1], Guangsha Shi[2], Gautam Gupta[3], Aditya Mohite[3], Swastik Kar[4], Emmanouil Kioupakis[2], Saikat Talapatra[1,5], K.M. Dani[1]*

[1] Femtosecond Spectroscopy Unit, Okinawa Inst. of Science & Technology, Graduate University, 1919-1 Tancha, Onna-son, Kunigami-gun, Okinawa, 904-0495 Japan.

[2] Department of Materials Science and Engineering, University of Michigan, 2106 H. H. Dow Bldg.,2300 Hayward St., Ann Arbor, MI 48109, United States

[3] Material Synthesis and Integrated Devices, MPA-11, Los Alamos National Laboratory, Los Alamos, NM 87545, USA

[4] Department of Physics, Northeastern University, Boston, Massachusetts 02115, United States

[5] Department of Physics, Southern Illinois University Carbondale, Carbondale, Illinois 62901, United States

Supplementary Information

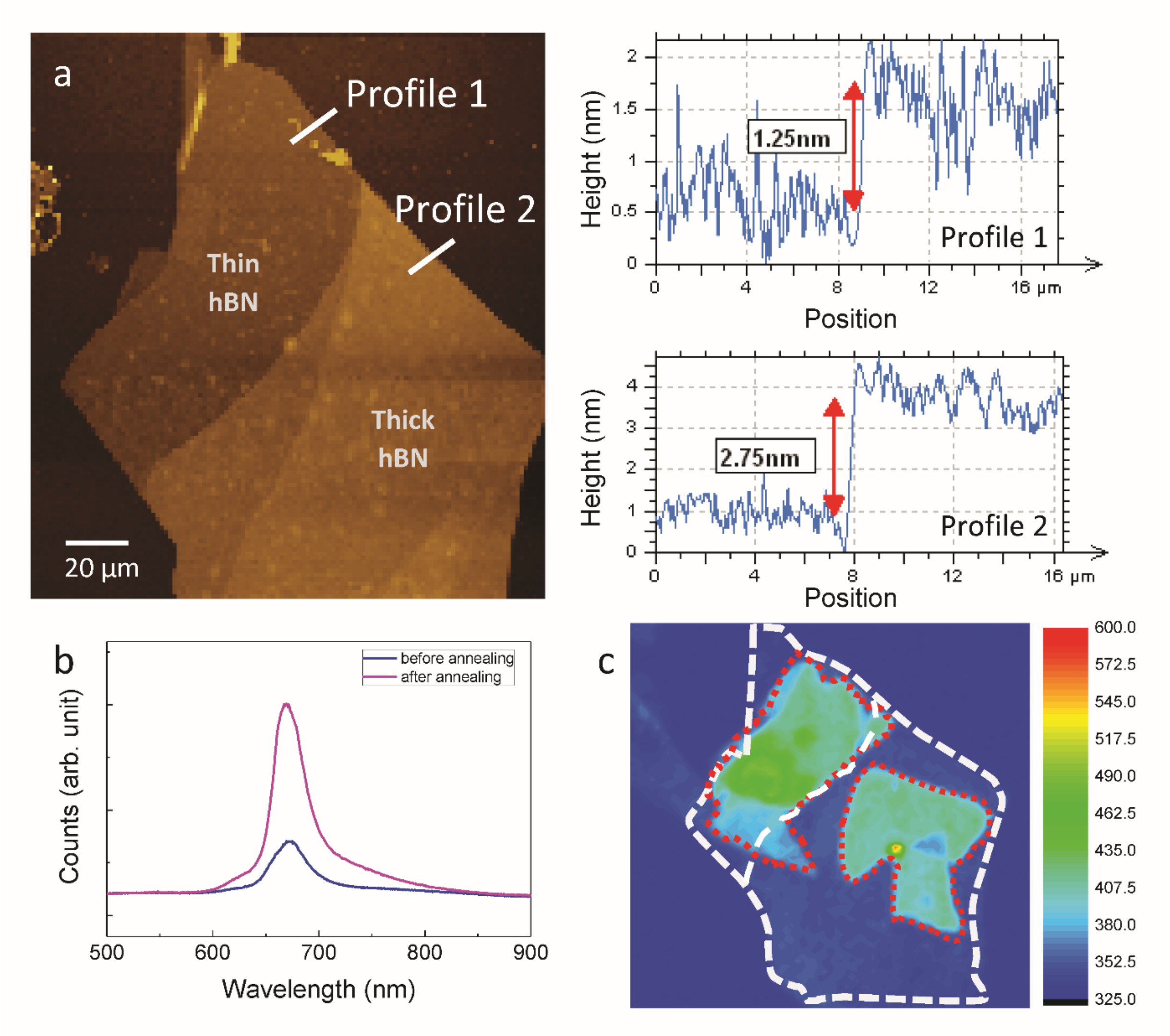

Supplementary Figure S1 | PL measurements for monolayer MoS_2 on top of an hBN buffer layer. a, AFM image of an hBN flake on top of a SiO_2 substrate. The line profile on the right shows that the thickness of hBN varied from 1.25nm to 2.75nm. **b**, shows the PL signal from monolayer MoS_2 flakes before and after the annealing-cleaning process. It shows that there is at least a 5x enhancement in the PL signal strength as a result of the annealing-cleaning process. **c**, shows a PL map of the MoS_2/hBN/SiO_2 heterostructure with monolayer MoS_2 (outlined in red) on top of the hBN flake (outlined in white). The thinner part of the hBN (top-left) is separated from the thicker part (bottom-right) by an extra dotted line in between. It shows that the PL signal strength for MoS_2 on hBN of different thickness is comparable, with MoS_2 on thinner hBN giving a slightly stronger signal, possibly due to interference effects.

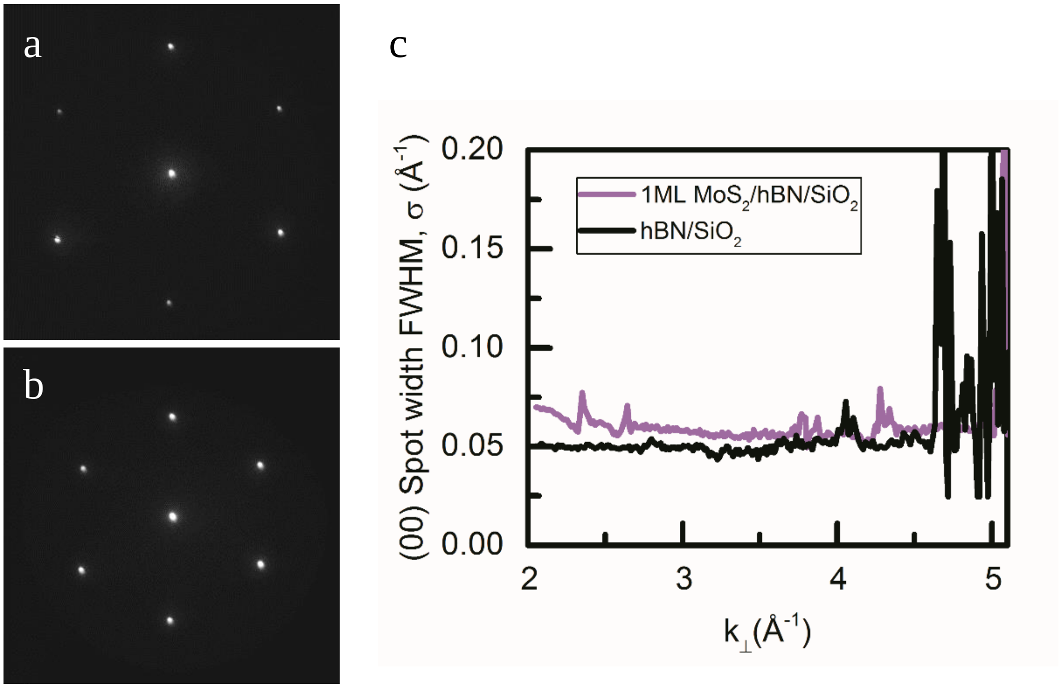

Supplementary Figure S2 | Flat hBN buffer on SiO_2. Sharp μ-LEED pattern are taken from **a,** hBN region as thin as ~1nm and **b,** monolayer MoS_2 on top of the thin hBN (electron energy at 50eV). **c,** FWHM of the specular (00) diffraction beam – σ, versus incident electron wave vector $k_\perp$ shows that both thin hBN and the MoS_2 on top are extremely flat. Thus, even atomically thin hBN on SiO_2/Si is able to provide a stable flat platform for an overlaying monolayer crystal.
